# High blood pressure readings on in-store machines: a qualitative study of the perspective of pharmacy staff

**DOI:** 10.1186/s40545-021-00297-9

**Published:** 2021-02-01

**Authors:** Ivona Mostarac, Clare L. Atzema

**Affiliations:** 1 Sunnybrook Research Institute, Sunnybrook Health Sciences Centre, Toronto, ON Canada; 2 Oakville Trafalgar Memorial Hospital Emergency Department, Halton Healthcare, Oakville, ON Canada; 3 York University, Toronto, ON Canada; 4 ICES, Toronto, ON Canada; 5 Division of Emergency Medicine, Department of Medicine, University of Toronto, Toronto, ON Canada; 6 Institute of Health Policy, Management and Evaluation, University of Toronto, Toronto, ON Canada

**Keywords:** High blood pressure, Hypertension, Pharmacy, Qualitative research

## Abstract

**Objectives:**

Emergency department (ED) visits for high blood pressure are on the rise. Yet the majority of these patients are discharged home after their ED evaluation, particularly those who present following an elevated reading on an in-store pharmacy machine. We aimed to gain insight on the practice and referral patterns of pharmacy staff who encounter a patient with an elevated in-store blood pressure (BP) reading.

**Methods:**

We conducted a qualitative study using semi-structured interviews with pharmacy staff (pharmacists and pharmacy technicians/assistants) from California, United States and Ontario, Canada. Interview questions were designed to examine the practice and referral patterns of pharmacy staff for patients with elevated in-store BP readings. Standard descriptive content analysis techniques were used to analyze the data and to develop themes for current practice and referral patterns.

**Results:**

Twenty-four interviews were completed: six with pharmacy technicians/assistants and 18 with pharmacists. Canadian pharmacy staff (83%) reported being approached frequently (defined as from weekly up to multiple times per day) by patients concerned about an elevated BP reading on an in-store machine, versus 50% reported by American participants. Participant definition of an elevated BP varied, with systolic values ranging from 120 to 150 mmHg and diastolic values from 60 to 90 mmHg. Participants emphasized the need to converse with and assess their patients prior to providing advice. The most frequently reported advice was to seek referral from an outside health care provider: ED, urgent care, or a primary care practitioner. Severity of the BP reading and symptomatology were reported as determining factors for referring patients to the ED. Pharmacists (92%) reported a lack of corporate and/or governing body policy for managing patients with in-store markedly elevated BP readings.

**Conclusions:**

Managing patients with an elevated BP reading in the community pharmacy setting is complex and not standardized. Referral to an external health care provider, including the ED, was a common theme. The development of a pharmacy referral tool/algorithm may be helpful to refer in-store patients with elevated BP readings to the most appropriate healthcare resources.

## Background

Hypertension (HTN) is a major contributor to the global chronic disease burden [1], affecting almost a quarter of the Canadian adult population [2] and nearly half of the American adult population [3]. The availability of self-monitoring blood pressure (BP) devices has increased since the early 2000s, and during the same time period, emergency departments (EDs) have seen a substantial rise in patients being seen for HTN [4–6]. In the United States (U.S.), the absolute number of ED visits for HTN increased by 30% between 2006 and 2012 [4]. Similarly, in Ontario, Canada, EDs saw a 64% increase in annual HTN ED visits between 2002 and 2012 [5]. Just under 10% of these visits are estimated to occur following an elevated BP reading at a pharmacy [6].

Our previous work suggests that many ED visits following an elevated BP reading at a pharmacy may not be necessary: the vast majority (> 97%) of these patients were discharged home at the end of their ED stay [6]. Moreover, among those who present following a pharmacy BP reading, mortality rates post-discharge are extremely low: 0% (95% confidence interval [CI], 0%–2.9%) mortality at two years [6]. In order to reduce ED visits by patients with elevated BP readings, however, it is critical to understand the process by which these patients end up at the ED.

Pharmacists play a vital role in the management of chronic diseases such as HTN [7–13]. Literature on BP management in the pharmacy setting has primarily addressed the validity of pharmacy BP readings [9], cost effectiveness of pharmacy interventions [10], pharmacist education and lifestyle advice [11], pharmacy/physician co-management of HTN [12], pharmaceutical management [11–13], and expansion of practice (i.e., allowing pharmacists to prescribe) [13]. To the best of our knowledge, no research has been conducted to address the perspectives of front-line community pharmacy staff on the management and referral of in-store patients with elevated BP readings.

The objective of this study was to use a qualitative approach to better understand the current practice of front-line Canadian and American community pharmacists and pharmacy technicians/assistants in the management and referral of patients with elevated in-store BP readings. A qualitative approach was chosen for its strength in analyzing data in an open-ended way, its ability to explore a new phenomenon that impacts decision-making, and the capacity to hear from new voices that are typically underrepresented in the literature (front-line community pharmacists and pharmacy technicians/assistants) [14].

## Methods

### Study design and setting

Using grounded theory methods, we conducted a qualitative study using semi-structured interviews with pharmacy staff, including pharmacists and pharmacy technicians/assistants, between August and September of 2019 in California, US and Ontario, Canada. Ontario and California are the most populous province and state in their respective countries [15, 16]. Both pharmacists and pharmacy technicians/assistants were selected to participate in our study, as patients seeking advice about an elevated BP reading could approach either individual at the pharmacy counter. This study was granted approval by the human research ethics board of Sunnybrook Health Sciences Centre in Toronto, Canada. Our process was in keeping with the COREQ [17] checklist and the Standards for Reporting Qualitative Research [18].

### Selection of participants, data collection, and processing

We applied purposive sampling when selecting cities in each province/state and interviews were completed in major cities (Toronto and Hamilton ON, San Diego CA), tourist cities (Niagara Falls and Niagara-on-the-Lake ON, Santa Barbara CA) and suburban cities (Burlington ON, Long Beach and Encinitas CA) (Table 1). Individual pharmacies that were a part of one of eight large pharmacy chains were chosen at random from the cities listed above. Inclusion criteria included consenting English-speaking pharmacists or pharmacy technicians/assistants who were employed by a large pharmacy chain in the selected cities/regions. We excluded pharmacists at independently owned pharmacies or pharmacies attached to a hospital, urgent care center, walk-in clinic, etc., because we aimed to investigate community pharmacy practice. Pharmacies with and without self-serve BP machines were included in our study, as patients can approach pharmacists for medical advice regarding an elevated BP reading taken at home using a pharmacy purchased home BP kit, and/or patients can ask the pharmacist for a consultation and manual BP check in-store.

**Table 1:** Study setting

Location, no. (%)	Canada (*n* = 12)	America (*n* = 12)
Major city	7 (58.3)	3 (25.0)
Toronto	4 (33.3)	0 (0)
Hamilton	3 (25.0)	0 (0)
San Diego	0 (0)	3 (25.0)
Tourist city	2 (16.7)	2 (16.7)
Niagara-on-the Lake	1 (8.3)	0 (0)
Niagara Falls	1 (8.3)	0 (0)
Santa Barbara	0 (0)	2 (16.7)
Suburban city	3 (25.0)	7 (58.3)
Burlington	3 (25.0)	0 (0)
Long Beach	0 (0)	3 (25.0)
Encinitas	0 (0)	4 (33.3)

Potential participants were approached in-person during off-peak (opening and closing) hours by the primary investigator (IM). They were notified that the interviewer was a prospective PhD student who was conducting hypertension research; participation and publication study consent information was discussed and consent for both was obtained (Appendix A and Appendix B). There were no established relationships with the study participants prior to study commencement. Study assumptions, biases, reasons, and interests in the research topic were not disclosed to the study participants.

The interview guide was developed by IM (ED nurse, York University clinical instructor, Masters trained in qualitative research, Sunnybrook Research Institute, previous experience conducting qualitative research) and CLA (Royal College certified emergency physician, Masters trained in clinical epidemiology, University of Toronto Associate Professor, ICES and Sunnybrook Research Institute Senior Scientist). The four questions were designed to obtain the pharmacy staff experience and perspective on managing patients with elevated BP readings in their pharmacies. The probing questions were designed to focus the participant on patient referral patterns and the rationale for those decisions. The interview guide was then reviewed and revised by the research team following twelve pilot tests. Once the study commenced, no changes were made to the interview guide. All interviews were conducted by IM (see Appendix A for interview guide). The preliminary analysis of the pilot tests revealed several themes that were used as a basis for deductive coding (IM, CLA). Reflexivity and researcher bias were addressed by the primary investigator recording her reactions and emotions in a personal research journal during the data collection and analysis [19].

Interview duration was approximately 5–10 min and interviews were conducted either at the pharmacy counter or in a private room intended for pharmacy consultations. Notes were taken by IM during and immediately after the interviews in order to ensure accuracy of information. The interviews were conducted in increments of six until no new concepts emerged and it was collectively determined that data saturation had been reached [20].

### Data analysis

The interview notes were transcribed verbatim into a Microsoft Excel File [Office 2016 for MAC] and then imported into NVivo-12 MAC [QSR, Doncaster, Australia] for analysis.

Standard descriptive content analysis techniques were applied in the data analysis process, in keeping with the standards for reporting qualitative research [18]. Drawing on grounded theory, codes were primarily developed deductively using the preliminary results from the pilot tests; additional codes were created inductively from within the study data. The interviews were conducted in sets of 6, the patterns and regularities were coded by two independent researchers (IM and CLA) and organized into themes and concepts. Any discrepancies were resolved via ongoing discussion amongst the research team members until consensus was reached. Interview conduction ceased once the research team was confident data saturation had been reached and no new themes emerged from a set of 6 interviews.

## Results

### Characteristics of study subjects

During the 2-month study period, 31 individuals were approached to participate in the study. Seven declined to participate: 5 stated that they were too busy, 1 stated that they were not interested, and 1 stated they were not able to participate. A total of 24 interviews were completed: Table 2 summarizes the characteristics of the study participants. Their reported length of practice ranged from 6 months to 42 years, with a mean of 9.6 (s.d. 11.3) years, 54% were female, and 75% were pharmacists.

**Table 2:** Participant characteristics

Characteristics, no. (%)	Canada (*n* = 12)	America (*n* = 12)
Sex		
Female	7 (58.3)	6 (50.0)
Professional designation		
Pharmacist	9 (75.0)	9 (75.0)
Pharmacy assistant/technician^a^	3 (25.0)	3 (25.0)
Years of practice		
< 1 year	0 (0.0)	1 (8.3)
1–5 years	7 (58.3)	6 (50.0)
6–10 years	1 (8.3)	3 (25.0)
> 10 years	4 (33.3)	2 (16.7)

^a^Technician in America. Assistant in Canada

### Main results

Most (83%; *n* = 10) Canadian participants reported being approached from weekly up to multiple times per day by patients concerned about an elevated BP reading on an in-store machine, while half (*n* = 6) of Americans reported the same. However, availability of in-store self-serve BP measurement devices varied between countries, with 100% (*n* = 12) of the Canadian pharmacies having self-serve machines compared to 67% (*n* = 8) of the American pharmacies. An additional 17% (*n* = 2) of American participants reported that while there was no self-serve BP machine in store, the pharmacist was able to check the patient’s BP with a manual machine upon request. The remaining 17% (*n* = 2) of American pharmacists noted while there was no self-serve machine, they were approached by patients concerned about an elevated BP reading taken elsewhere.

**Fig. 1:**
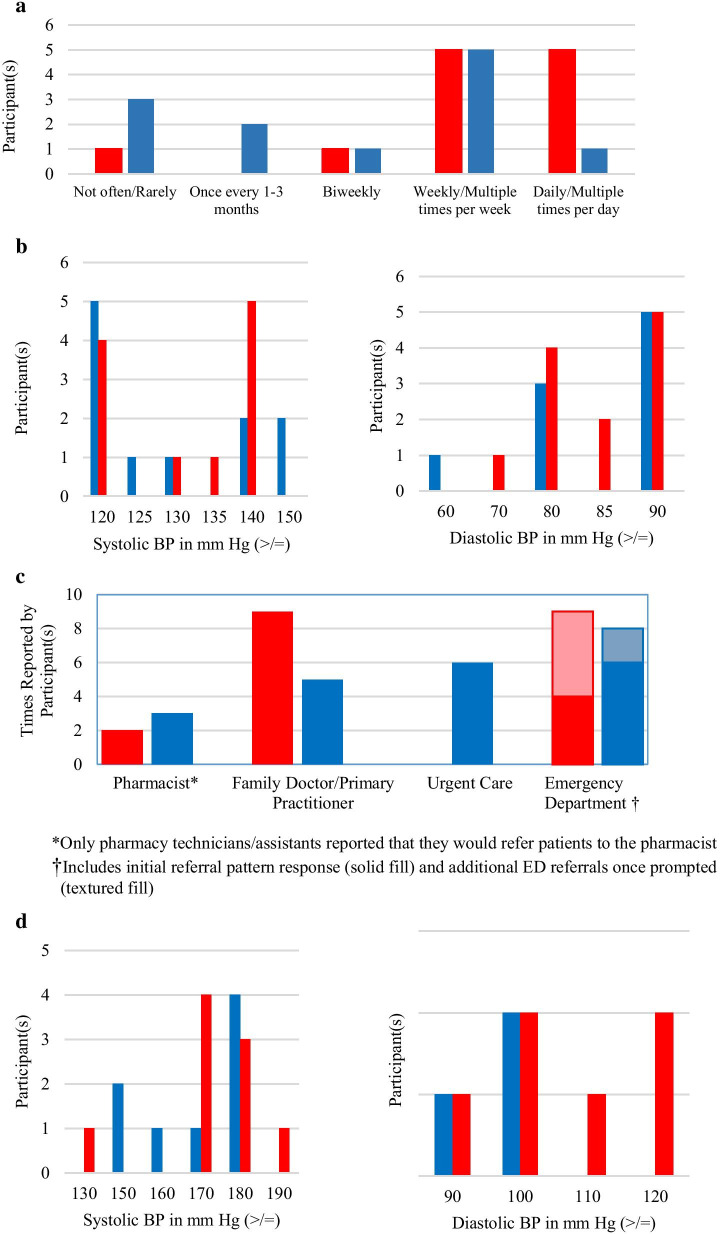
Comparisons in practice between Canadian (red) and American (blue) pharmacists and pharmacy technicians/assistants. **a** Reported frequency of concerned customers seeking advice regarding elevated in-store BP readings. **b** Definition of a high BP. **c** Referral patterns. **d** Reported BP values that would lead to a referral to the ED

Participants reported variable definitions of “high” BP, with systolic values ranging from 120–150 mmHg and diastolic values ranging from 60 to 90 mmHg (Fig. 1). The most frequent systolic BP value reported as high by Canadian participants was ≥ 140 mmHg (46%), while for Americans it was ≥ 120 mmHg (46%). Both Canadians (42%) and Americans (56%) most frequently reported a diastolic value of ≥ 90 mmHg as high.

Pharmacists in both countries made statements with similar frequency about referring patients to the ED (Canada 9 quotes; America 8 quotes). Out of the 9 Canadian and 8 American ED quotes, 4 Canadian and 6 American participants reported their referral of choice is the ED, with an additional 5 Canadian and 2 American participants reporting that they either have referred patients to the ED in the past or that they would refer in the future if they deemed necessary. On the contrary, urgent care and primary care referral patterns varied between the two countries, with American pharmacy staff making 6 references to urgent care while Canadians made no references to urgent care (Fig. 1). Moreover, Canadians reported referring their patients to a primary care practitioner (8 quotes) more frequently than their American counterparts (5 quotes). Of note, 5 of the 6 pharmacy technicians/assistants reported that they would not refer a patient to an outside healthcare provider, as all referrals would be passed to the staff pharmacist at their respective pharmacy.

The qualitative analysis of the open-ended responses is summarized in Table 3 with representative quotations to support the outlined themes and subthemes. Six primary themes arose from the interview data: (1) definition and importance of high BP is not based on the BP value alone; (2) pharmacists converse with the patient and assess the situation prior to providing advice; (3) advice provided is patient-specific and dependent on various patient factors; (4) advice/interventions provided are multifaceted, (5) no store/governing body policy is available for pharmacists to follow when providing advice to patients with elevated in-store BP readings, and (6) referral to the ED is dependent on several factors.

**Table 3:** Themes, subthemes and supporting quotations from pharmacists and pharmacy technicians/assistants about their experiences and practice with customers who are concerned about an elevated blood pressure reading

Theme	Subtheme	Quotation
Definition of high BP is not based on the BP value alone	Demographics	No value, background. There is a formula to screen for hypertension and pre-hypertension. Age, history but it is hard to do in retail 120/80 is the average but it fluctuates between gender, age, weight. Depends on the person
	Comorbidities	Depends if they don’t have any diabetes or kidney disease. If no diabetes 140/90 or if diabetic, have kidney disease or heart disease it needs to be under 130/80
	Guideline used	Depends on which guidelines you follow. ADA guidelines look at diabetes, age. They say 140/90 for diabetics. If not ASCVD risk, if healthy 150/90 but I don't go through everything. ASCVD, JNC7, could follow those too. Controversial topic, diabetes linked to hypertension
	BP consistently elevated	Depends…if it is a first reading or if it is always above 140/90
Pharmacists will converse with the patient and assess the situation prior to providing advice	Troubleshoot (encourage to relax and retake BP)	First I try and calm them down, try to retake their blood pressure in 5 min Sometimes I manually check, calm them down, retake it I tell them to relax and retake it. They could be sort of pacing, sit for 10 min then go to the machine Have another reading. Running around, stress makes it go up sometimes
	Review events prior to blood pressure taking	Did you walk? Have coffee? Did they just exercise or drink coffee? Because that will elevate it. The reading is just a snapshot of that moment
	Discuss lifestyle (diet/exercise)	Before the readings I discuss their diet and lifestyle
	Review medical history and medications/medication compliance	I go over their diagnosis, medications, their history Ask about medications, sometimes medications need to be added Some have high blood pressure medications, are they using any blood pressure medications? Are they taking medications as prescribed?
	Assess symptoms	They have a headache sometimes. Go through all of that I ask about symptoms, headache, that is concerning
Advice is patient specific and dependent on various patient factors	BP reading/value	Changes with whatever reading they have. If above 170 to ER. If it is lower, I give them pharmacy and non-pharmacy advice Check with doctor unless it is really elevated then urgent care Depending on where they stand…If it is quite high, I advise them to see their family doctor or go to urgent care. If it's not that high, come back and retake it in the afternoon
	BP consistently elevated	He said 150/90 [BP reading], I said that's fine but if consistently high go to the doctor If it is high all the time, I tell them to go to their doctor
	Symptoms	Check up with family doctor if they do not feel well, tired
	Medications/history of HTN	Tell them to follow up with their doctor if medications need to be changed It depends [advice]. Are they on blood pressure medications?
Advice/interventions provided are multifaceted	Reassurance/education	A lot of times it is 130 or 120 and they freak out. That happens often…[I] provide hypertension education, what is high Tell them caffeine intake can increase blood pressure if they had it a half an hour before. A full bladder can raise it too Anything over 135 is high on the machine so they come running. “No man, it isn't high. You're fine” The machine is not as accurate as the doctor's office. The cuff size is standard here…Blood pressure is dynamic, not just one reading
	Monitor BP/keep logbook	Tell them to keep a log of their readings Record blood pressure a few times Monitor it
	Lifestyle changes (diet/exercise)	I tell them what they can do to lower it, decrease salt, increase exercise I tell them about how to change their diet and exercise Do they eat salty food or any canned food? Tell them to avoid it
	PRN medication administration	Some have blood pressure medications, fast acting that they take if their blood pressure is high. We tell them to take it
	Seek medical attention	I advise them to see their family doctor or go to urgent care It is better to speak with a doctor to be monitored Make an appointment with your doctor
No store/governing body policy for pharmacists to follow when providing advice to patients with elevated in-store BP readings	Professional knowledge used to guide advice	No policy but what I know from my profession, my knowledge No [policy], just professional judgement and obligation. If they have an elevated reading and ran out of medications I have to record their blood pressure in record if refilling blood pressure medication and I have to call the prescriber
	Familiarity/comfort with patient guides advice	Regular patients, look at their history, medications. If not a regular patient [I] send them to urgent care to get a check up No [policy], individualized, how much I know them. How many times they have taken it in a row [BP]? Do they have high blood pressure?
	Follow a HTN guideline	[I] follow current guidelines but there are 3. There is no in-store policy per se, they just want us to follow one of the guidelines No, not really [policy]. Guidelines you can follow though
	Advice is pharmacist specific, have their own set of rules/guidelines	No, all pharmacists have their own. You may get different advice from different pharmacists No policy. Set of rules to follow. No exercise, sitting down, no speaking, rest, no caffeine for a half hour to 1 hour before taking blood pressure No policy. Just ask about any medications, conditions, allergies. Nothing specific [in terms of policy]
Referral process to the ED is dependent on several factors	Symptoms/presentation	Symptoms. Dizzy, not able to walk, tired, headache If they feel their heart beat higher, you have to consider heart attack. If they are tired or don’t feel well Someone grabbing their chest saying “I am having a stroke” I would think those patients go straight to the emergency department Experiencing any other symptoms, lightheaded and dizzy Symptoms, feel dizzy, feel like having a headache, tend to ask to go to emergency…have a buzzing in their ears Headache, vision change, I suggest to go to the emergency department. See a doctor right away
	Severity of BP value	One time it was 200/140 so I said “do you need me to call 911 for you?” Over 180 or 190 to emergency department or urgent care 180 plus or diastolic well over 100 [to ED] If really really elevated, stroke level…I don't know how much you know about hypertension but not everyone has symptoms when their reading is high so it depends. Someone with a high reading could be having a stroke Doctor's office unless it is over 180, then go to the hospital. That is an emergency Cut-off 200s. Straight to emergency, or maybe 190, close to 200. They need to see a doctor sooner than later
	BP consistently elevated	If it is consistently high go to emerge, it is always there
	Factors potentially contributing to elevated reading	Gather more information. They missed a dose [medication]? Had caffeine or walked far? Just talk to them
	Access to/availability of a primary care practitioner	Urgent care or emergency department if they don't have a primary doctor Call their prescriber, their regular doctor and see if they can order a one-time dose of something. I've had that happen before too. But if not, and over 180 or 190 to emergency department or urgent care
	Presence of comorbidities	Sometimes it is their blood glucose reading. Like if it is over 300, call 911
	No past medical history of HTN	If they don’t have a history [HTN] then definitely tell them they need to get it checked out now [urgent care or ED]
	Weekday vs. weekend	One case around 180–190, called the ambulance, it was a weekend, I couldn't let her go
	Familiarity with patient/access to drug profile	If I have their profile, I will change their medication dose [increase it] and tell them to see their doctor [as opposed to advising them to go to the ED]

### Definition and importance of high BP is not based on the BP value alone

Participants reported that the BP value alone cannot be used to determine if the patient has a “high” BP: 4 other components need to be considered to put the value in context. The definition of high BP is dependent on the presence or absence of (1) comorbidities such as diabetes, renal and cardiovascular disease (8 quotes), with participants reporting a lower threshold for high BP in patients with comorbidities; (2) the guideline the pharmacist/pharmacy technician applies (4 quotes) was also reported as relevant. Participants referred to the following guidelines: American Diabetes Association (ADA), Atherosclerotic Cardiovascular Disease (ASCVD) Primary Prevention Guideline, Seventh Report of the Joint National Committee on Prevention, Detection, Evaluation, and Treatment of High Blood Pressure (JNC 7), Hypertension Canada and the Canadian Hypertension Education Program (CHEP). Other reported factors that were considered included the following: (3) patient demographics such as age, sex, and weight (4 quotes) and (4) whether the patient’s BP is consistently elevated, or if this was a one-time high reading (1 quote).

“Depends on if they have diabetes. If it is a first reading or if it is always above 140/90 for non-diabetics and 130/80 for diabetics.”

“Depends on which guidelines you follow. ADA guidelines look at diabetes, age. They say 140/90 for diabetics. If not ASCVD risk, if healthy 150/90 but I don't go through everything. ASCVD, JNC7, could follow those too. Controversial topic, diabetes is linked to hypertension.”

### Pharmacists will converse with the patient/assess the situation prior to providing advice

Pharmacists expressed an obligation to engage the patient and assess the situation prior to providing any advice. This included 5 key subthemes: (1) troubleshooting with the patient/encouraging them to relax and retake their BP (10 quotes); (2) discussing the patient’s medical history and their medications/medication compliance (7 quotes); (3) reviewing activities prior to BP taking that may influence an elevated reading such as exercise, caffeine, and stress (5 quotes); (4) discussing diet and exercise habits (2 quotes) and (5) assessing the patient for any symptoms (2 quotes).

“A lot of times it is 130 or 120 and they freak out. That happens often. 'Did you walk? Have coffee?' Sometimes I manually check, calm them down, retake it. Gather more information, ask background questions.”

“It depends [advice]. Are they on blood pressure meds? Tell them they need to sit and rest before the reading. Did they just exercise or drink coffee because that will elevate it, the reading is just a snapshot of that moment.”

### Advice is patient specific and dependent on various patient factors

The advice provided by pharmacists to patients with an elevated blood pressure varied based on factors that have been broken down into 4 subthemes. Pharmacists reported taking into account (1) if the patient had a history of HTN/were on anti-hypertensive medication(s) (8 quotes), (2) their BP value (8 quotes), (3) if their BP was consistently elevated (3 quotes) and (4) if they had any symptoms with their elevated BP reading (4 quotes). Patients who had a history of HTN/were on anti-hypertensive medication(s), had consistently elevated readings, and/or were symptomatic were more likely to be referred to an outside healthcare provider (primary care practitioner, urgent care, ED).

“30–40 above target [target BP defined as 140/90] with symptoms is concerning. Headache, vision change I suggest to go to the emergency department. See a doctor right away, may need a medication change.”

### Advice and interventions provided are multifaceted

The advice and/or interventions pharmacists provided to their in-store hypertensive patients was categorized into 5 subthemes. Advising patients to (1) seek medical attention from outside healthcare providers (primary care practitioner, urgent care, ED) was the most frequently reported intervention, with 18 coded quotes. Other reported advice/interventions included (2) keeping a logbook/monitoring BP readings (10 quotes), (3) providing reassurance/education (8 quotes), (4) recommending lifestyle changes such as increasing exercise and decreasing sodium intake (4 quotes) and taking a (5) PRN (as needed) anti-hypertensive medication (1 quote).

“I tell them about how to change their diet and exercise. Tell them to follow up with their doctor if medications need to be changed. Tell them to keep a log of their readings.”

### No store/governing body policy for pharmacists to follow when providing advice

A lack of policies both from the governing body and the store/corporation of the pharmacy was noted by pharmacists in 14 coded quotes. Only 1 American pharmacist reported a corporate policy that included an information sheet on HTN.

“[organization] has a blood pressure screening sheet, 130/80 is uncontrolled, [I] forward the sheet to their physician”

In turn, pharmacists reported 4 tools that guide/help their advice: (1) their professional knowledge (4 quotes), (2) their own personal set of rules/guidelines (3 quotes), (3) HTN guidelines (1 quote) and (4) their familiarity/comfort with the patient (1 quote).

### Referral process to the ED is dependent on several factors

Participants reported referring patients to the ED was a complex decision involving many factors that were broken down into 9 subthemes. (1) Severity of the BP value was the most frequently reported factor, with 43 coded quotes. The reported systolic and diastolic values that triggered referral to an ED ranged from 130–190 mmHg, and 90–120 mmHg, respectively (Fig. 1); Canadians most frequently reported a systolic value of 170 mmHg (44%) and diastolic values of 100 (40%) and 120 (40%) mmHg or greater as warranting a referral to the ED, while Americans reported a systolic value of 180 mmHg (50%) and diastolic value of 100 mmHg (67%) or greater. (2) Symptoms/patient presentation was reported as the second most important factor guiding the ED referral decision-making process, with 16 coded quotes. Reported symptoms that result in ED referral include: dizziness/lightheadedness, vision changes, tinnitus, headache, chest pain, palpitations, weakness and feeling generally unwell. Other reported concerning findings that would warrant referral to the ED included the following: 3) the BP being consistently elevated; 4) lack of availability/access to a primary care practitioner; 5) suboptimal day of the week (weekend vs weekday); 6) lack of familiarity with the patient/access to their drug profile; 7) presence of other comorbidities such as diabetes; 8) no past medical history of HTN and 9) no external factors that could explain the elevated BP such as a missed medication dose, caffeine intake and exercise.

“Very elevated to emergency department, especially if symptomatic.”

## Discussion

This study is the first to explore the practice, referral patterns, and rationale for referrals for patients with elevated in-store BP readings, as reported by front-line pharmacy staff. Canadian pharmacy staff reported being approached more frequently by patients concerned about an elevated BP reading in comparison to American pharmacy staff, which was likely secondary to more self-serve BP machines at the Canadian pharmacies in our study. Both stated that the ED was a common choice for referral of patients with an elevated BP for whom they had a clinical concern. The reported BP threshold for ED referral varied widely, with systolic values ranging from 130 to 190 mmHg and diastolic values from 90 to 120 mmHg. Similarly, reported symptoms that increased the likelihood of an ED referral varied from relatively benign symptoms such as tinnitus and feeling generally unwell to potentially emergent symptoms such as chest pain, headache, weakness, dizziness/lightheadedness, and vision changes. Most pharmacists (92%) reported no in-store or governing body policy for them to follow when providing advice and/or referrals for hypertensive patients.

The importance of the pharmacist’s role within the healthcare system in hypertension management is well documented [7–13, 21]. Pharmacists are more available to the public for consultation compared to primary care practitioners, and they have the opportunity to dedicate more face-to-face time with their patients (on average 30–60 min compared to 15 min with a primary practitioner) [7, 8, 21]. Pharmacist involvement in medication management, hypertension education, and lifestyle counseling has been illustrated to have positive health outcomes for patients with hypertension [11–13, 21]; however, we found that pharmacists reported a lack of direction from their regulatory body and/or their employer on how to manage the frequently encountered patients with elevated BP readings in their pharmacies. At present, the Canadian pharmacy practice regulatory body mandates that decisions and recommendations made by pharmacists in practice be based on an evidence-informed approach and that accurate explanations be provided for decisions made, without specific directives for patients with elevated BP readings [22]. In California, the Pharmacy Lawbook contains a specific sections (4103) entitled, *Blood Pressure—Taking by Pharmacists*, which suggests that pharmacists may take a patient’s blood pressure, inform them of the reading, interpret the results (within a high, low, or normal range), and advise them to seek medical attention from a provider of *the patient’s* choosing [23]. Therefore, decision-making around referral is potentially left to the patient and not the provider. Future collaborative research between pharmacists, primary care, emergency medicine providers, and patients, among others, is needed to standardize the pharmacy referral process, and in turn improve the lack of coordination between healthcare disciplines, which currently often operate in silos.

Our study found American pharmacists report that they refer patients with elevated BP readings to 1 of 3 health care avenues (ED, urgent care, or a primary care practitioner), while Canadian pharmacists only reported making referrals to the ED and primary care. Urgent care clinics in Canada and the U.S. can vary from small stand-alone clinics with extended hours that are typically run on a walk-in basis by one or many family physicians, to large centers that are stand-alone EDs that may provide specialty services, including laboratory testing and imaging [24–27]. The lack of reported referrals to urgent care by Canadian pharmacists is likely due to the divergent healthcare system structure between the two countries, including a general lack of urgent care centers in the Canadian healthcare system. Urgent care centers were first implemented in the 1980s in both countries; however, the expansion of urgent care centers varied greatly, with the U.S. estimating between 12 and 20 thousand urgent care centers nationwide in 2007, while Canada only estimated 25 centers in the year 2000 [24, 27]. Finally, emergency visits are expensive for Americans, while urgent care can offer similar services at a fraction of the price [28, 29]. The ability to pay is an issue that Canadian pharmacists may not consider when referring patients, as Canadians do not pay out-of-pocket for healthcare services due to Canada’s universal healthcare system [30].

In turn, Canadian participants reported making more referrals to primary care practitioners compared to their American counterparts. Canadian pharmacists may have been more inclined to refer to primary care as only 7.5% of Ontarians aged 12 years or older lack access to a regular physician, compared to 22% of adult Americans who lack access [31, 32]. Moreover, 41% of Canadians are able to obtain same-day or next day access to their primary care provider, and in general Ontarians report high primary healthcare access scores, suggesting they are satisfied with their perceived access [33, 34].

A myriad of intersecting factors influenced the choice to refer for ED care, including BP level, use of anti-hypertensives, comorbidities and symptomology, to name a few. Published work on the perspectives of emergency physicians in elevated BP management demonstrates similarities to what we found [35]. Higher BP levels and the presence of more comorbidities increased the odds of an ED physician prescribing or increasing the dose of an existing antihypertensive prescription, which is in keeping with our study findings of higher BP readings resulting in ED referrals [35]. In our study, participants most frequently reported SBP values from 170 to 180 mmHg and DBP value of 100 mmHg to be of concern and therefore warrant a referral to the ED: similarly, another study found that half of ED physicians reported that they would initiate or increase outpatient antihypertensive therapy in an asymptomatic patient at a median SBP of 200 mmHg and a median DBP of 110 mmHg [35]. Moreover, both studies noted the approach to managing hypertension is dependent on the presence of comorbidities, with the number of comorbidities playing an important role into the decision to initiate or alter an antihypertensive treatment, a similar finding to our study with comorbidities being considered a vital factor in the decision-making process for ED referrals [35]. The similarities in criteria suggest that physicians and pharmacists can easily work together to establish an integrated approach to managing hypertensive patients across healthcare settings.

The reported definition of high BP varied between Canadian and American participants, with Canadian participants most frequently reporting a systolic reading of > 140 mmHg and American participants a systolic reading of > 120 mmHg. The variation may be explained by the recent divergence between American and Canadian hypertensive guidelines, and thus differing goals for target BP between the two countries. Based on the Systolic Blood Pressure Intervention Trial (SPRINT) study [36], the American Heart Association changed their definition of elevated blood pressure in 2017 to a SBP of 120–129 mmHg and Stage I HTN to a SBP of 130–139 mmHg [37]. In contrast, while Canadian guidelines acknowledge the SPRINT study, and encourage physicians to discuss lower SBP targets with their patients, they continue to apply the more liberal value of > 140/90 mmHg to define HTN [2, 38, 39]. Regardless of the level chosen, it is clear that participants were up to date with their respective country’s HTN guidelines.

## Limitations

Our study is not without limitations. The data were collected over a short period of time and our sample size was small; however, it was sufficient for qualitative work, where the goal is for significant themes to emerge from the data (and saturation was met). Many interviews were conducted at the pharmacy counter and on occasion interviews were interrupted and later resumed due to the participant needing to attend to a patient. The primary investigator aimed to minimize interruptions by conducting the interviews during non-peak hours and by using the pharmacy consultation room to conduct interviews when possible. Transcript review after the fact was not possible in our study as no personal or identifiable information was collected from participants and immediate review post-interview was challenging as participants had to tend to patients. To counter this, the primary investigator verbally confirmed responses when possible. Interviews were conducted by one researcher, the primary investigator, which may be construed as a limitation, however since this was an unfunded study it was not possible to hire an independent interviewer. The primary investigator was adequately trained in qualitative research methodology, has previous experience in conducting qualitative research and all interview notes were reviewed by a coauthor (CLA) and consensus for emerging themes was established. Our results cannot be quantitatively generalized, as our interview guide did not include specific patient scenarios for participants to analyze and report their practice patterns; that was not the goal of this qualitative study. Finally, while our study was conducted in two countries, the results may not highlight themes that might have arisen in other populations, including rural settings, as the scope of practice of pharmacists may vary not only from country to country but state to state.

## Conclusions

Our study indicates that managing patients with acutely elevated BPs in the community pharmacy setting is complex, and typically performed in the absence of a guideline or consensus tool for direction. Pharmacists and technicians reported incorporating multiple factors during an extended assessment. Following that assessment, they reported frequently referring patients to the ED for care, where other work suggests that 97% of these patients will be discharged home from the ED. The development of a consensus pharmacy referral tool or guideline via collaboration between pharmacists, family medicine, emergency medicine specialists, and patients may help to standardize the in-store management of patients with elevated BPs, and offer patients the most appropriate healthcare services.

## Data Availability

The datasets used and/or analyzed during the study are available from the corresponding author on reasonable request.

## Appendices

### Appendix A. Interview guide

Hello, my name is Ivona Mostarac, and I am a prospective PhD student conducting hypertension research with my supervisor, Dr. Clare Atzema. My goal is to publish the findings in a medical journal. Could I have a moment of your time to ask you a few questions regarding your practice? With your permission, I will be taking down notes to help me keep track of our conversation. However, I won’t be collecting or keeping anything that can identify you—not your name or anything else, just your responses. I will leave you an information sheet to look over as well.

If agrees:

May I confirm your professional designation, are you a pharmacist or pharmacy technician? How long have you been practicing? Thank you.

**Table:**

	Interview questions	Rationale/explanation
1	Do customers approach you because they are concerned about an elevated blood pressure reading after using the in-store blood pressure machine? Probes: How often would you say this happens?	Introduction to the topic of self-monitoring blood pressure devices in pharmacies. Open-ended question which allows for dialog This question is meant to get a sense of how often this is occurring in practice, if at all
2	How do you define high blood pressure? Probes: Is there a specific value that you use?	Definition of HTN varies depending on the resource used. This question is trying to assess which guidelines pharmacists are using when providing education and advice (if they are using a guideline at all) This probe is trying to zero in on a specific cut-off blood pressure value
3	What advice do you provide to these customers? Probes: Is there a policy or standardized approach that you use?	This question is meant to explore the current practice of pharmacists with this specific patient population Meant to assess if there are discrepancies between pharmacies. Are there specific policies or procedures which guide the pharmacist’s response?
4	Where do you refer customers with an elevated blood pressure? Probes: Do you ever refer customers to the emergency department? What factors do you consider when referring to the emergency department? Is there a cut-off blood pressure value that you use in order to make this decision?	The question starts off open-ended to see what healthcare resource comes to mind first (family practitioner, ED, urgent care, telehealth, etc.) The probe questions try to narrow down if pharmacists ever refer customers to the ED specifically. If so, which information do they use in order to make this decision? Is there a specific cut-off blood pressure value which results in a referral to the ED?

## Abbreviations

ED: Emergency department
BP: Blood pressure
HTN: Hypertension
US: United States
COREQ: Consolidated Criteria for Reporting Qualitative Research
ON: Ontario
CA: California
ADA: American Diabetes Association
ASCVD: Atherosclerotic Cardiovascular Disease
JNC 7: Primary Prevention Guideline, Seventh Report of the Joint National Committee on Prevention, Detection, Evaluation, and Treatment of High Blood Pressure
CHEP: Canadian Hypertension Education Program
